# Effects of latitude and depth on the beta diversity of New Zealand fish communities

**DOI:** 10.1038/s41598-017-08427-7

**Published:** 2017-08-14

**Authors:** Vincent Zintzen, Marti J. Anderson, Clive D. Roberts, Euan S. Harvey, Andrew L. Stewart

**Affiliations:** 1 Department of Conservation Te Papa Atawhai, National Office, 18-32 Manners St, PO Box 10 420, Wellington, 6143 New Zealand; 2 New Zealand Institute for Advanced Study (NZIAS), Massey University, Albany Campus, Auckland, New Zealand; 3 Museum of New Zealand Te Papa Tongarewa, 169 Tory Street, Wellington, New Zealand; 4 Department of Environment and Agriculture, Building 311 Room 208, Curtin University, GPO Box U1987, Perth, WA 6845 Australia

## Abstract

Marine ecosystems are difficult to sample quantitatively at increasing depth. Hence, few studies attempt to measure patterns of beta diversity for ecological communities in the deep sea. Here we (i) present and quantify large-scale gradients in fish community structure along depth and latitude gradients of the New Zealand EEZ, (ii) obtain rigorous quantitative estimates of these depth (50–1200 m) and latitudinal effects (29.15–50.91°S) and their interaction, and (iii) explicitly model how latitudinal beta diversity of fishes varies with depth. The sampling design was highly structured, replicated and stratified for latitude and depth, using data obtained from 345 standardised baited remote underwater stereo-video deployments. Results showed that gradients in fish community structure along depth and latitude were strong and interactive in New Zealand waters; latitudinal variation in fish communities progressively decreased with depth following an exponential decay (*r* ^2^ = 0.96), revealing increasingly similar fish communities with increasing depth. In contrast, variation in fish community structure along the depth gradient was of a similar magnitude across all of the latitudes investigated here. We conclude that an exponential decay in beta diversity *vs* depth exists for fish communities present in areas shallower than the New Zealand upper continental slope.

## Introduction

Understanding spatial patterns of biodiversity along large-scale global gradients, such as latitude, altitude and depth, is a primary goal in ecology^1–3^. Such knowledge is key to the development of principles for sound environmental management^4^. In the terrestrial environment, latitudinal and altitudinal biodiversity gradients are well-characterised^5, 6^, but there are far fewer ecological studies quantifying patterns of marine biodiversity *vs* depth, due to logistic difficulties associated with sampling and observing marine systems, especially at increasing depths^7^. Most marine studies of fish diversity have been limited to narrow geographic^8^ or depth ranges^9^, or rely on museum or web-based records of species distributions that are incomplete^10, 11^. Some attempts at synthesizing patterns of species richness and zonation in the deep sea have been made^7, 11–13^, but studies of global-scale patterns of beta diversity in the deep sea are absent for the megafauna and rare for other taxa^14^. Exploratory field-based studies at larger scales, especially for the deep sea, tend to have sampling designs that are opportunistic or unstructured (where depth, latitude and longitude are measured as covariates)^15–18^ or have incommensurable sampling units (e.g., trawls of varying duration or of varying types)^13, 16–20^. Consequently, most studies are not directly amenable to drawing broader scientific inferences without certain caveats or the use of additional modelling or standardization techniques to account for such issues^21, 22^.

Early ecological studies on land suggested that observed changes in community structure with increasing latitude (i.e., latitudinal beta diversity) mirror changes seen with increases in elevation; hence generating interactions in their effects on biodiversity^23, 24^. As early as 1956, the hypothesis that faunal communities are wide-spread and broadly connected with large-scale patterns of oceanic circulation was put forward^25^. This idea was further supported by distributional studies of North Atlantic^26^ and southeastern Australian fishes^27^. Later, studies in the ocean also showed possible interactive effects of latitude and depth on fish communities; more specifically, assemblages become more similar to one another with increasing depth, reducing differences in fish communities observed along latitudinal gradients. For example, increased similarity in megafaunal assemblages with depth was reported from New England and Cape Hatteras^28, 29^, and similar patterns were more recently depicted for fishes^21, 30–32^.

Here, we describe results from a large-scale study of the biodiversity of fishes in New Zealand waters having a sampling design that was carefully structured with respect to two factors: latitude (7 locations spanning 29.15–50.91°S) and depth (7 strata spanning 50–1200 m). Spatial stratification, replication and the use of a standardized size of sampling unit ensured the validity of the study design for drawing rigorous quantitative inferences. The essential aims of our study were: (1) to partition the variation in fish community structure (beta diversity) according to latitude, depth and their interaction; (2) to rigorously estimate latitudinal beta diversity separately for each depth stratum, with estimates of its variability; and (3) to model explicitly how latitudinal beta diversity of fishes varies with depth. This study presents patterns on beta diversity resulting from the interaction between latitude and depth, while most studies in the past have focussed on presenting changes in species identities along the depth gradient only, without reference to any horizontal spatial component^7, 14^.

## Results

**Figure 1:**
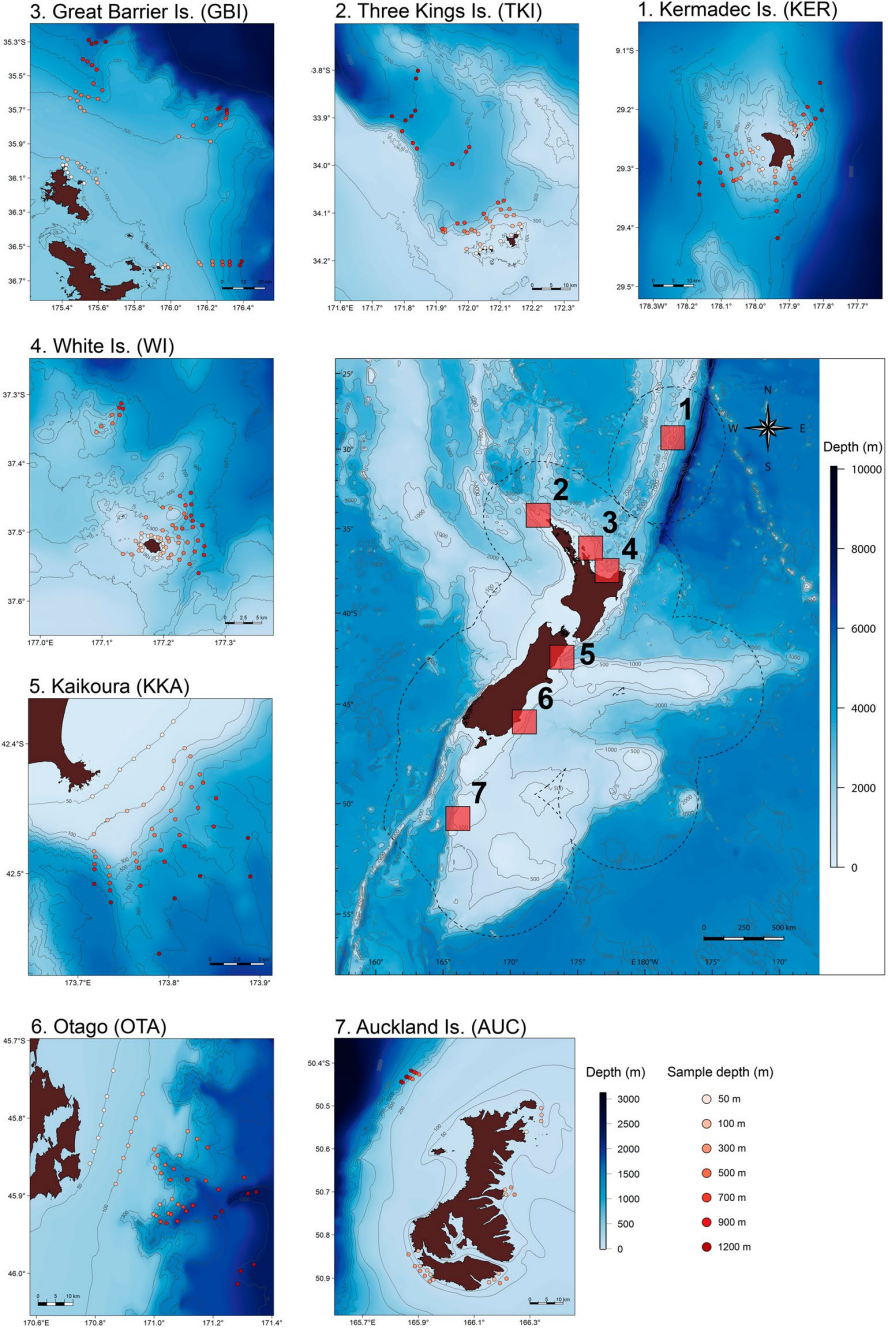
Latitudinal beta diversity sampling sites. Fishes were sampled at multiple depths (50, 100, 300, 500, 700, 900 and 1200 m) using baited remote underwater stereo-video (stereo-BRUVs) deployments at each of seven locations: Kermadec Islands, Three Kings Islands, Great Barrier Island, White Island, Kaikoura, Otago and the Auckland Islands. Map created using R^85^. Bathymetry source: Depth contour polyline (Hydro, 1:350 k–1:1,500 k), Land Information New Zealand, Crown Copyright Reserved. Land data source: NZ Coastlines (Topo, 1:50 k), Land Information New Zealand, Crown Copyright Reserved.

**Figure 2:**
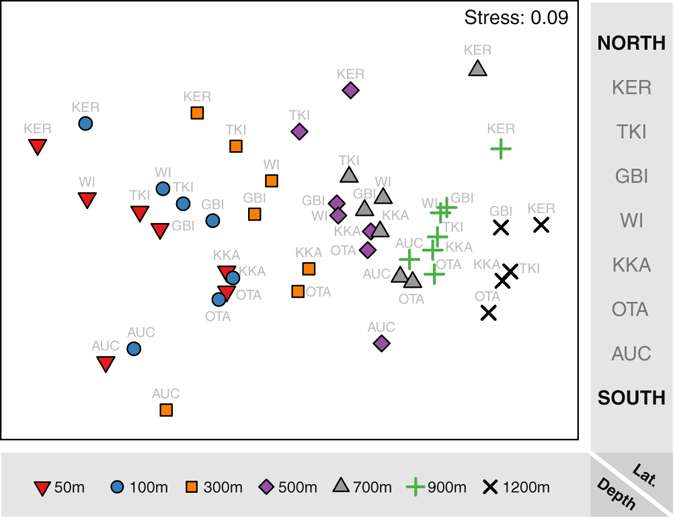
Ordination analysis of fish communities by depth and latitude. Non-metric multi-dimensional scaling (nMDS) ordination plot of Jaccard dissimilarities among fish assemblages consisting of depth-by-location averages (centroids) from a minimum of *n* = 6 video deployments (except for the Auckland Islands and White Island where no deployments were made at 1200 m). Each centroid is identified by a symbol to indicate the depth (50, 100, 300, 500, 700, 900 or 1200 m) and a label to indicate the location (from north to south: Kermadec Islands (KER), Three Kings Islands (TKI), Great Barrier Island (GBI), White Island (WI), Kaikoura (KKA), Otago (OTA) and the Auckland Islands (AUC)).

From 345 video deployments (Fig. 1), a total of 242 fish taxa belonging to 166 genera in 84 families and 25 orders were identified (Supplementary Tables S1 and S2, and Supporting Video S3; see also Supplementary Figure S4 for species accumulation curves per depth stratum). The composition of fish assemblages was strongly structured by depth (i.e., from shallow to deep along nMDS axis 1, Fig. 2) and by latitude (i.e., from south to north along nMDS axis 2, Fig. 2). There was also a clear and highly statistically significant interaction in fish community structure between latitude and depth (Fig. 2 and Table 1). Fish communities became progressively more similar to one another with increasing depth, as indicated by an increased clustering of points associated with the different locations for deeper strata, especially at 900 m and 1200 m (Fig. 2). The largest component of variation was that associated with the residual (42.2% of the total), followed by the depth-by-location interaction (24%) and the main effects of depth (17.2%) and location (12.4%), respectively, while individual transects (variation among sites within the same location) accounted for comparatively little (3.8%, Table 1).

**Table 1:** PERMANOVA partitioning of fish assemblages on the basis of Jaccard dissimilarities in response to Location (Lo, fixed, 7 levels), Depth (De, fixed, 7 levels) and Transects (Tr, random, nested within Lo) using Type I (sequential) sums of squares.

Source	df	MS	Pseudo-*F*	*P*	Sqrt(Component of variation)	%
Lo	6	34947	8.486	0.0001	25.5	12.4
De	6	45295	20.235	0.0001	30.0	17.2
Tr(Lo)	70	3068	1.386	0.0001	14.2	3.8
Lo × De	33	10806	4.882	0.0001	35.8	24.4
Residual	225	2200			47.0	42.2
Total	340					

*P*-values were obtained for each term in the model using 9999 permutations under a reduced model. Estimated sizes of effects for each term in the model are shown in Jaccard units as the square-root of the *pseudo* multivariate component of variation. Variation attributable to each term in the model is also expressed as a percentage of the total (%). Note that 4 of the original 345 video deployments were omitted prior to analysis, as there were no fish recorded in them.

**Figure 3:**
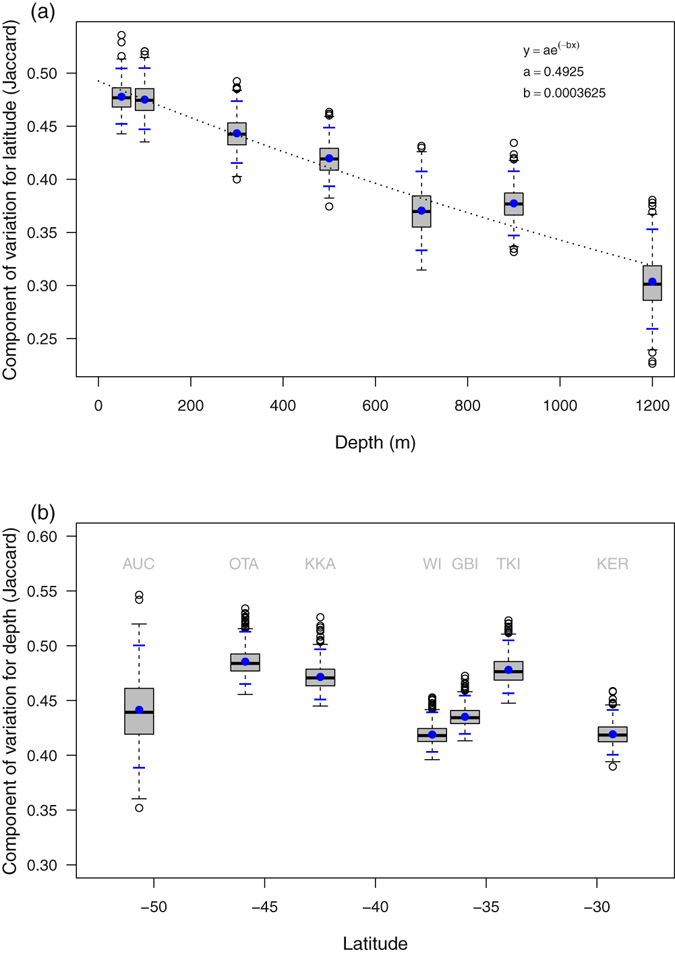
Large-scale beta diversity of fishes versus depth and latitude. Relationship between (**a**) component of variation within each depth stratum (a surrogate for latitudinal beta diversity) *vs* depth; and (**b**) component of variation within each location (vertical beta diversity) *vs* latitude, based on square-root-transformed *pseudo* multivariate components of variation calculated from a PERMANOVA partitioning of Jaccard dissimilarities (blue dots). Box plots in grey show distributions of values from 1000 bias-corrected separate-sample bootstraps of residuals. Blue dashes show 95% confidence intervals based on the 0.025 and 0.975 quantiles of the bias-corrected bootstrap distributions. The dotted line in (**a**) shows the **e**xponential model of the decay in latitudinal beta diversity *vs* depth.

The source of the significant depth-by-location interaction arises from a decrease in latitudinal variation with increasing depth, from 50 to 1200 m (Fig. 3a). Although this relationship could be modelled with a simple linear regression (*r* ^2^ = 0.97), a more appropriate (and highly statistically significant) ecological model is an exponential decay (*r* ^2^ = 0.96, *P* = 0.0001, Fig. 3a); variation in the structure of fish assemblages is not expected to reach an absolute value of zero and then to become negative, as a linear model would imply. In contrast, there was no clear relationship between the estimated component of variation for depth *vs* latitude (*r* ^2^ = 0.12, *P* = 0.445, Fig. 3b). Thus, variation in fish community structure along the depth gradient was of a similar magnitude across all locations. The estimated component of variation for the residuals (i.e., variation among individual deployments within a given combination of depth-by-location) showed no consistent relationship with either depth or location (*P* = 0.461 and *P* = 0.168, respectively).

Furthermore, the exponential decay in latitudinal beta diversity versus depth appeared not to be driven by patterns in either mean alpha or gamma diversity alone, whose patterns along the depth gradient each varied in different ways at different locations (see Supplementary Figure S5).

## Discussion

Here we have provided the first direct quantitative model of latitudinal beta diversity at large spatial scales in the ocean along a depth gradient, calculated rigorously from a carefully stratified, standardized replicated sampling design. Previous studies based on observational data were not sufficiently standardized to allow direct explicit quantification of these effects for modelling purposes^21, 30, 31^.

Most previous studies of beta diversity *vs* depth have focused specifically on how species’ identities, abundance or biomass changes from one depth stratum to another, particularly to identify patterns of zonation^12, 14, 18, 33–35^, or to quantify overall turnover *vs* depth^18, 21, 32^. Our study provides the first attempt to rigorously model the general increase in similarity of fish assemblages with increasing depth at broad spatial scales (i.e, along a large latitudinal span, not just along a depth gradient). This has also been called horizontal beta diversity^14^.

The parsimonious model we propose indicates strongly that the effects of latitude diminish exponentially with depth. This model of exponential decay might hold more generally for other marine taxa, such as benthic megafauna^28, 36^, molluscs^37^ or asteroids^38^, which all show a general pattern of increased similarity in large-scale comparisons with increasing depth based on unstructured observational data. More recently, a distance decay relationship between depth and Bray-Curtis similarities between samples of molluscs was found in the Atlantic Ocean^14^, based on previously published data^37^. Habitats in deep environments are more homogeneous than those in shallower environments^39, 40^. They are also geologically more stable, cover a greater geographic area and are biogeographically more connected than are habitats in shallower systems^41, 42^. These patterns contrast strongly with those found for terrestrial habitats at high elevations, which are environmentally highly variable, are smaller in area, less geologically stable and are geographically fragmented compared to low-elevation habitats^43, 44^. Hence, observed decreases in beta diversity *vs* depth in the ocean will require different mechanistic explanations to those that may be advanced to explain decreasing beta diversity *vs* elevation in terrestrial studies.

Our observations are consistent with the hypothesis that circulation of large-scale water masses influences species’ distributions^26, 27^. This hypothesis is based on the broad extent of intermediate and deep water masses^45^ which mirrors the large biogeographic ranges of many deep-sea species. Five waters masses are important around New Zealand^46^: the surface subtropical and Subantarctic Waters, the Antarctic Intermediate Water, the Deep Water and the Antarctic Bottom Water. The two surface water masses meet to form a front called the Subtropical Convergence which approximately follows the 15 °C surface isotherm in summer and the 10 °C surface isotherm in winter and the 34.7–34.8 × 10^−3^ surface salinity isopleth^47^. These two water masses have very different origins and characteristics (high *vs* low salinity). The complex front created by their collision creates an increased diversity of potential niches for species. In addition, the Subantarctic Front, which separates Australasian Subantarctic Water from Circumpolar Subantarctic Water, is also likely to influence the southern locations of this study. In contrast, the entire latitudinal range we sampled is bathed, at depths starting at 700–1000 m, by a unique water mass, the Antarctic Intermediate Water. Although a degree of temporal and spatial variability in the properties of Antarctic Intermediate Waters exists^48^, the relative homogeneity of this water mass may explain a decrease in latitudinal beta diversity of fishes. Indeed, our results showed a marked decrease in beta diversity at 700–900 m, in agreement with the expected position of this water mass.

In addition to the “water mass” hypothesis, several other potential mechanistic explanations could explain these observed patterns of beta diversity along the depth gradient. For example, energy can play an important role in structuring patterns of biodiversity^49^. Particulate organic carbon (POC) that sinks from shallower waters is the main source of energy for assemblages occurring at depths in the ocean >200 m, beyond the photic zone. The flux of POC is seasonal and also decreases with depth following a power law; it is estimated that less than 10% of the carbon produced in shallow waters reaches depths >2000 m^50^. POC availability appears to be a very important determinant in structuring communities from local to global scales^14^. A relatively homogeneous distribution of generalist wide-ranging species may present an optimal evolutionary strategy to optimize biological intake of scarce energy resources, yielding reduced large scale horizontal beta diversity at depth.

Another important feature of increased depth is the increasing pressure, which can have strong effects on the molecular structure of proteins; specialized physiological mechanisms are required to stabilize protein structures at deep depths^51^. Levels of trimethylamine oxide (TMAO) that may help to stabilize proteins under high pressure increase linearly with depth in teleosts^52^. Thus, TMAO content may set the depth limit for teleosts at *ca*. 8,500 m, where fish cells may become hyperosmotic to seawater^53^; it might also more generally limit the speciation of fishes with increasing depth. Although reduced species’ diversity in itself does not necessarily explain reduced beta diversity, narrow available niche space would allow a small number of successful species to become widely distributed across a spatially homogeneous and highly pressurized habitat, resulting in reduced beta diversity.

The exponential decay model for beta diversity *vs* depth proposed here has important implications for our understanding of ecological biodiversity in the deep sea. Extrapolation of the model, although clearly speculative, suggests that the percentage of unshared species among fish assemblages becomes very small (i.e., *ca*. 10% unshared and 90% shared) at depths beyond 4400 m. This coincides with the abyssal sea floor – vast plains at 3,000–5,000 m depth that represents the most common benthic environment on the planet, covering 54% of the Earth’s surface^54^. Obviously, there are multiple factors that might come into play at depths greater than 1200 m, including influences of water masses^26, 27^ and surface-water productivity^55, 56^. However, the relative homogeneity of abyssal environments is consistent with past observations indicating that species inhabiting these habitats have broad distributional ranges^25, 26, 41, 42^. Indeed, as early as 1880, Henry N. Moseley, in his report of the *Challenger* expedition stated: “There is absolutely nothing to restrict the geographical range of animals in the deep sea. We got tired on the Challenger of dredging up the same monotonous animals wherever we went”^57^.

Deep-sea research, in general, and spatially-structured and replicated deep-sea data, in particular, are still far too depauperate, however, to address many fundamental hypotheses, which remain to be tested. Further research exploring wider depth ranges at other locations and latitudes is warranted to explore the generality of the exponential decay model of fish beta diversity *vs* depth. In addition, the ecological and evolutionary drivers of changes in beta diversity with increasing depth may be clarified in future studies by comparing and contrasting patterns in taxonomic, phylogenetic and functional beta diversity at large scales^44, 58, 59^.

New knowledge gained from future studies regarding the physical, historical and biological drivers of beta diversity in the ocean must be integrated with known effects of human-mediated disturbances, such as pollution, habitat modification, and fishing. Indeed, a further implication of our results is that conservation efforts will require an extra level of sophistication in shallow-water systems, which not only tend to be highly impacted by human activities, but are also the richest and show the greatest variation and turnover of marine fauna. Establishment of marine reserves at remote, deep, offshore locations may be valuable for deep-sea ecosystem functioning, but will provide little-or-no protection or compensation for degradation of habitat and rapid species loss in coastal environments. Multi-faceted large-scale assessments of the biodiversity of earth’s oceans^59^ will serve our ultimate aim for ecosystem knowledge, resource management and sustainability.

## Methods

### Locations and sampling methods

Baited remote underwater video (BRUVs) imagery was collected from 2009–2012, but always during the austral summer months, at each of seven locations: the Kermadec Islands (KER), the Three Kings Islands (TKI), Great Barrier Island (GBI), White Island (WI), Kaikoura (KKA), the Otago Peninsula (OTA) and the Auckland Islands (AUC), spanning a latitudinal range of 21° in New Zealand waters (Fig. 1 and Table 2). At each location, video samples were generally taken along each of at least *n* = 6 replicate transects at each of seven targeted depths: 50, 100, 300, 500, 700, 900 and 1200 m. However, logistic constraints and poor weather led to some imbalance in the study design (Table 2). A sonar was used to deploy units at the desired depth, achieving average depths (±SD) for the different target strata of 51 m (±8.6), 101 m (±9.7), 304 m (±21.5), 505 m (±24.3), 699 m (±32.6), 894 m (±40.5) and 1170 m (±41.0), respectively. The resulting dataset comprised a total of 345 deployments. Individual deployments were separated by at least 500 metres (usually by much more, see Fig. 1) and remained on the seabed for a minimum of two hours (only the first two hours of each deployment are considered in this study).

**Table 2:** Range in latitude and longitude for deployments obtained at each of the seven locations and the number of baited remote underwater stereo-video (stereo-BRUVs) deployments obtained at each depth within each location.

Location (date of sampling)	Latitude range Longitude range	Sample size							
		50 m	100 m	300 m	500 m	700 m	900 m	1200 m	Total
Kermadec Islands (KER) (05/2011)	29.4176°S–29.1549°S	7	7	7	7	7	7	7	49
	181.8354°E–182.1948°E								
Three Kings Islands (TKI) (03/2010)	34.1851°S–33.8010°S	8	6	6	7	6	6	6	45
	171.7616°E–172.1666°E								
Great Barrier Island (GBI) (12/2009)	35.2893°S–36.6235°S	8	9	8	8	6	8	8	55
	175.3588°E–176.3911°E								
White Island (WI) (03/2009)	37.5595°S–37.3128°S	11	12	11	11	9	11	0	65
	177.0911°E–177.2663°E								
Kaikoura (KKA) (11/2010)	42.5900°S–42.3829°S	7	7	7	7	7	7	7	49
	173.7161°E–173.9983°E								
Otago peninsula (OTA) (02/2012)	46.0136°S–45.7387°S	7	7	7	7	7	7	7	49
	170.7819°E–171.3465°E								
Auckland Islands (AUC) (02/2012)	50.9114°S–50.4181°S	14	9	2	2	3	3	0	33
	165.8385°E–166.3412°E								
Total		62	57	48	49	45	49	35	345

The stereo-video units consisted of two HD Sony handycams in underwater housings mounted on a base bar 0.7 m apart and inwardly converged at 8°. Bait consisted of 2 kg of chopped pilchards (*Sardinops sagax*) held in bait bags that were visible in the cameras’ field of view. Lighting was provided by eight blue LED lamps, each delivering a radiant flux of 340–425 mW at wavelengths of 450–465 nm. Sensors attached to the frame of the units also recorded the depth, temperature and salinity of each deployment *in situ*. See Zintzen *et al*.^32^ for further technical details regarding the stereo-BRUV apparatus. The use of BRUVs provided a standardized sampling methodology *in situ* across the full study design and across the full range of potential habitats, capturing images of a wide spectrum of fishes, not just piscivores (see Supplementary Tables 1 and 2). For further details regarding pros and cons associated with the use of BRUV methodology, see Harvey *et al*.^60^ and related refs 61, 62.

### Image analysis

Analysis of video footage was performed following Zintzen, *et al*.^32^. The maximum number of individuals (MaxN) of the same species appearing within five meters of the unit in a video frame was used as a conservative estimate of the relative abundance of fish seen from each two-hour stereo-BRUV deployment^63, 64^. At the beginning and completion of the field work, the stereo-BRUVs were calibrated following procedures outlined in Harvey and Shortis^65^ and Shortis and Harvey^66^ using Cal software (v1.32, www.seagis.com.au). Calibration was also done at the completion of fieldwork to ensure that the positions of stereo units remained fixed over the course of all deployments. Importantly, stereo (bi-camera) systems ensure a standardized field of view (volume ~24.6 m^3^) for rigorous estimation of relative abundance and composition across the study design, as well as 3D perception for observing behaviour and movements for an extensive variety of fishes, alive and *in situ* ^63^.

### Fish identification

Accurate species identification made solely from camera images is often difficult, particularly when the fish fauna is poorly known taxonomically. In our study, identification errors have been greatly reduced through (i) obtaining very high definition digital images from top quality BRUV equipment; (ii) using forward-facing BRUV system cameras to provide taxonomically essential lateral images of fishes (cf. downward-facing views, commonly used for benthos in deep sea systems); (iii) large fish traps deployed at the same time, depth range, and location as the video systems, to capture voucher specimens for identification and registration into the Te Papa National Fish Collection and used to underpin and inform most of the video identifications; (iv) all laboratory and most video identifications being carried out by world experts, who were preparing new descriptions and keys for all 1262 known fishes known within the EEZ^67^; (v) difficult identifications were made conservatively; fishes not identifiable to species were named to their lowest taxonomic level possible. As a result, 191 out of the 242 taxa recorded (79%) were identified to species level. Analyses done using only fish identified to species level did not change the fundamental results and inferences.

### Multivariate partitioning of variation in community structure (beta diversity)

To quantify biodiversity along depth (or altitude), direct measures of spatial variation based on standardized units must be obtained and compared for different depths (or altitudes). Given an appropriately structured and replicated sampling design, a multivariate partitioning of the variation in community structure (i.e., beta diversity)^68^ due to large-scale factors (such as depth, latitude and their interaction) can be quantified explicitly^69, 70^, and the relative sizes of these components of variation can be directly compared^71, 72^. This general and new approach has never been applied before to any large-scale study of beta diversity from a structured sampling design in either marine or terrestrial systems and is described in the following sections.

### Statistical analysis

Although counts of each fish species (MaxN) were available, we focused on presence/absence (i.e., compositional) information only. All analyses were based on the Jaccard dissimilarity measure^73^), which is directly interpretable as the proportion of unshared species between two sampling units out of the total number of species present in both sampling units. It is also a measure of beta diversity^74^ and can optionally be expressed as a percentage (i.e., ×100). The matrix correlation between the dissimilarity matrix based on Jaccard *vs* that based on Bray-Curtis^75^, a measure widely used in ecology that includes relative abundances^76^, was very large (Spearman’s rho = 0.987).

To visualise patterns of dissimilarities among fish communities across the study design, non-metric multidimensional scaling ordination (nMDS)^77^ was done on Jaccard dissimilarities calculated on averages for each species within each combination of depth-by-location (*n* ≥ 6 deployments per cell). The full set of data at the replicate level was analysed using permutational multivariate analysis of variance (PERMANOVA^69, 78^) according to the following three factors: Location (fixed with 7 levels), Depth (fixed with 7 levels) and Transect (random, nested in Location, with varying numbers of transects per Location).

Although the design was unbalanced (Table 2), multivariate analogues to traditional univariate expectations of mean squares can be used to construct correct *pseudo F*-ratios (i.e., where the numerator and denominator have the same expectation under a true null hypothesis) and to obtain appropriate tests by permutation for each term in the model, using the software PERMANOVA+^70^, an add-on to PRIMER v7^79^. These analyses were done using Type I (sequential) sums of squares and *P*-values were obtained using 9999 permutations under a reduced model^80, 81^. Results were not affected in any way by changing the order of terms in the sequential analysis.

Components of variation for each term in the model were calculated using ANOVA estimators, obtained by setting mean squares equal to their expectations and solving for the parameters of interest^82^. When calculated from a PERMANOVA model, these should generally be thought of as “pseudo” multivariate components of variation^70^, as they do not include estimation of covariances, and they can be based on a dissimilarity measure of choice (in our case, Jaccard). These components of variation are direct measures of beta diversity^74^, and are additive in the sequential PERMANOVA model, so can also be expressed as a percentage of the total variation across the study design. Furthermore, when these components are transformed by taking square roots, they are then in the units of the original dissimilarity measure (e.g., Jaccard units), and provide a direct dissimilarity-based multivariate analogue to a standard deviation. Thus, for example, a square-root-transformed component of variation with a value of 20 for the factor of Latitude would mean that the standard deviation of a given location centroid from the overall centroid is approximately 20 Jaccard units (i.e., 20% unshared species).

A statistically significant location-by-depth interaction (see Results) indicated that latitudinal variation in fish community structure differed for different depth strata. Multivariate *pseudo* variance components, as measures of beta diversity for a given spatial scale, can be calculated along one or more environmental gradients (e.g., see mission statement V4(b) depicted in Fig. 4 of Anderson *et al*.^74^). Thus, a component of variation for latitude was estimated separately within each depth stratum, and *vice versa*. Bootstrap resampling algorithms (including appropriate bias corrections) can be implemented to provide empirical confidence intervals around such estimates for statistical inference^72^. Empirical 95% confidence intervals for each estimated component were obtained from 1000 bias-corrected separate-sample bootstraps of residuals^83, 84^. These analyses were done using purpose-built R code^85^. A general function in R (“comp.var”) for estimating *pseudo* multivariate components of variation for one factor (factor A) within each level of another factor (factor B) on the basis of the Jaccard measure, including bootstrap confidence intervals with empirical bias-correction, is provided as Supporting Information (Supplementary Method S6).

### Data availability statement

The datasets generated during and/or analyzed during the current study are available from the corresponding author on reasonable request.

## Electronic supplementary material

Supplementary Information

Supplementary Video S3
