# Supplementary material for: Effects of latitude and depth on the beta diversity of New Zealand fish communities
Source: Sci Rep. 2017 Aug 14;7:8081. doi: 10.1038/s41598-017-08427-7 (PMC5556088; doi:10.1038/s41598-017-08427-7)
Supplement: Supplementary file 1 — Supplementary Information [file 41598_2017_8427_MOESM1_ESM.pdf]

Effect of latitude and depth on the beta diversity of New Zealand fish communities

SUPPLEMENTARY INFORMATION

Vincent Zintzen, Marti J. Anderson, Clive D. Roberts, Euan S. Harvey, Andrew L. Stewart

SUPPLEMENTARY TABLE S1. List of species from two-hour baited remote underwater videos (stereo-BRUVS) deployed at seven depth strata at each of seven locations in New Zealand waters. Locations: Kermadec Islands (KER), Three Kings Islands (TKI), Great Barrier Island (GBI), White Island (WI), Kaikoura (KKA), Otago (OTA) and the Auckland Islands(AUC). Depth strata: 50, 100, 300, 500, 700, 900 and 1200m.

Taxa	Level of identification	Family	Order	Class
Alepocephalidae sp.	Family	Alepocephalidae	Osmeriformes	Actinopterygii
Alepocephalus australis Barnard, 1923	Species	Alepocephalidae	Osmeriformes	Actinopterygii
Allocyttus niger James, Inada & Nakamura, 1988	Species	Oreosomatidae	Zeiformes	Actinopterygii
Allocyttus sp.	Genus	Oreosomatidae	Zeiformes	Actinopterygii
Amblyraja cf.hyperborea (Collett, 1879)	Species	Rajidae	Rajiformes	Chondrichthyes
Amphichaetodon howensis (Waite, 1903)	Species	Chaetodontidae	Perciformes	Actinopterygii
Anampses sp.	Genus	Labridae	Perciformes	Actinopterygii
Antimora rostrata (Günther, 1878)	Species	Moridae	Gadiformes	Actinopterygii
Apristurus sp.	Genus	Scyliorhinidae	Carcharhiniformes	Chondrichthyes
Arripis xylabion Paulin, 1993	Species	Arripidae	Perciformes	Actinopterygii
Aulacocephalus temmincki Bleeker, 1857	Species	Serranidae	Perciformes	Actinopterygii
Bassanago bulbiceps Whitley 1948	Species	Congridae	Anguilliformes	Actinopterygii
Bathygadus cottoides Günther, 1878	Species	Macrouridae	Gadiformes	Actinopterygii
Bathypterois longifilis Günther, 1878	Species	Ipnopidae	Aulopiformes	Actinopterygii
Bathyraja shuntovi Dolganov, 1985	Species	Arhynchobatidae	Rajiformes	Chondrichthyes
Benthodesmus sp.	Genus	Trichiuridae	Perciformes	Actinopterygii
Beryx decadactylus Cuvier in Cuvier & Valenciennes, 1829	Species	Berycidae	Beryciformes	Actinopterygii
Beryx splendens Lowe, 1833	Species	Berycidae	Beryciformes	Actinopterygii
Bodianus flavipinnis Gomon, 2001	Species	Labridae	Perciformes	Actinopterygii
Bodianus unimaculatus (Günther 1862)	Species	Labridae	Perciformes	Actinopterygii
Bovichtus sp.	Genus	Bovichthyidae	Perciformes	Actinopterygii
Brotulotaenia nigra Paulin & Roberts, 1989	Species	Ophidiidae	Ophidiiformes	Actinopterygii
Bythaelurus dawsoni (Springer, 1971)	Species	Scyliorhinidae	Carcharhiniformes	Chondrichthyes
Caesioperca lepidoptera (Forster, 1801)	Species	Serranidae	Perciformes	Actinopterygii
Callanthias australis Ogilby, 1899	Species	Callanthiidae	Perciformes	Actinopterygii
Callanthias sp.	Genus	Callanthiidae	Perciformes	Actinopterygii
Callorhinchus milii Bory de St. Vincent, 1823	Species	Callorhinchidae	Chimaeriformes	Chondrichthyes
Canthigaster callisterna (Ogilby, 1889)	Species	Tetraodontidae	Tetraodontiformes	Actinopterygii
Caprodon longimanus (Günther, 1859)	Species	Serranidae	Perciformes	Actinopterygii

**Taxa**	**Level of identification**	**Family**	**Order**	**Class**
Capromimus abbreviatus (Hector, 1875)	Species	Zeniontidae	Zeiformes	Actinopterygii
Carcharhinus galapagensis (Snodgrass & Heller, 1905)	Species	Carcharhinidae	Carcharhiniformes	Chondrichthyes
Carcharhinus sp.	Genus	Carcharhinidae	Carcharhiniformes	Chondrichthyes
Carcharodon carcharias (Linnaeus, 1758)	Species	Lamnidae	Lamniformes	Chondrichthyes
Centriscops humerosus (Richardson, 1846)	Species	Macroramphosidae	Syngnathiformes	Actinopterygii
Centroberyx affinis (Günther, 1859)	Species	Berycidae	Beryciformes	Actinopterygii
Centrophorus harrissoni McCulloch, 1915	Species	Centrophoridae	Squaliformes	Chondrichthyes
Centrophorus sp.	Genus	Centrophoridae	Squaliformes	Chondrichthyes
Centroscymnus coelolepis Bocage & Capello, 1864	Species	Somniosidae	Squaliformes	Chondrichthyes
Centroscymnus macracanthus (Regan, 1906)	Species	Somniosidae	Squaliformes	Chondrichthyes
Centroscymnus owstoni Garman, 1906	Species	Somniosidae	Squaliformes	Chondrichthyes
Centroselachus crepidater (Bocage & Capello, 1864)	Species	Somniosidae	Squaliformes	Chondrichthyes
Cephaloscyllium isabellum (Bonnaterre, 1788)	Species	Scyliorhinidae	Carcharhiniformes	Chondrichthyes
Cephaloscyllium sp.B	Species	Scyliorhinidae	Carcharhiniformes	Chondrichthyes
Cepola haasti Hector, 1881	Species	Cepolidae	Perciformes	Actinopterygii
Chaetodon guentheri Ahl, 1923	Species	Chaetodontidae	Perciformes	Actinopterygii
Chauliodus sloani Bloch & Schneider, 1801	Species	Stomiidae	Stomiiformes	Actinopterygii
Cheilodactylus francisi Burridge, 2004	Species	Cheilodactylidae	Perciformes	Actinopterygii
Cheilodactylus spectabilis (Hutton, 1872)	Species	Cheilodactylidae	Perciformes	Actinopterygii
Chelidonichthys kumu (Lesson & Garnot, 1826)	Species	Triglidae	Scorpaeniformes	Actinopterygii
Chimaeridae sp.	Family	Chimaeridae	Chimaeriformes	Chondrichthyes
Chromis cf.abyssicola Allen & Randall 1985	Species	Pomacentridae	Perciformes	Actinopterygii
Chromis dispila Griffin, 1923	Species	Pomacentridae	Perciformes	Actinopterygii
Chromis sp.	Genus	Pomacentridae	Perciformes	Actinopterygii
Cirrhigaleus australis White, Last & Stevens, 2007	Species	Squalidae	Squaliformes	Chondrichthyes
Coelorinchus acanthiger Barnard, 1925	Species	Macrouridae	Gadiformes	Actinopterygii
Coelorinchus aspercephalus Waite, 1911	Species	Macrouridae	Gadiformes	Actinopterygii
Coelorinchus biclinozonalis Arai & McMillan, 1982	Species	Macrouridae	Gadiformes	Actinopterygii
Coelorinchus bollonsi McCann & McKnight, 1980	Species	Macrouridae	Gadiformes	Actinopterygii
Coelorinchus fasciatus (Günther, 1878)	Species	Macrouridae	Gadiformes	Actinopterygii
Coelorinchus innotabilis McCulloch, 1907	Species	Macrouridae	Gadiformes	Actinopterygii
Coelorinchus kermadecus Jordan & Gilbert in Jordan & Starks, 1904	Species	Macrouridae	Gadiformes	Actinopterygii
Coelorinchus mycterismus McMillan & Paulin, 1993	Species	Macrouridae	Gadiformes	Actinopterygii
Coelorinchus mystax McMillan & Paulin, 1993	Species	Macrouridae	Gadiformes	Actinopterygii

**Taxa**	**Level of identification**	**Family**	**Order**	**Class**
Coelorinchus oliverianus Phillipps, 1927	Species	Macrouridae	Gadiformes	Actinopterygii
Coelorinchus sp.	Genus	Macrouridae	Gadiformes	Actinopterygii
Colistium nudipinnis (Waite, 1910)	Species	Pleuronectidae	Pleuronectiformes	Actinopterygii
Colistium sp.	Genus	Pleuronectidae	Pleuronectiformes	Actinopterygii
Conger verreauxi Kaup, 1856	Species	Congridae	Anguilliformes	Actinopterygii
Congiopodus sp.	Genus	Congiopodidae	Scorpaeniformes	Actinopterygii
Coris picta (Bloch & Schneider, 1801)	Species	Labridae	Perciformes	Actinopterygii
Coris sandeyeri (Hector, 1884)	Species	Labridae	Perciformes	Actinopterygii
Coryphaenoides murrayi Günther, 1878	Species	Macrouridae	Gadiformes	Actinopterygii
Coryphaenoides rudis Günther, 1878	Species	Macrouridae	Gadiformes	Actinopterygii
Coryphaenoides serrulatus Günther, 1878	Species	Macrouridae	Gadiformes	Actinopterygii
Coryphaenoides subserrulatus Makushok, 1976	Species	Macrouridae	Gadiformes	Actinopterygii
Cyttus novaezealandiae (Arthur, 1885)	Species	Cyttidae	Zeiformes	Actinopterygii
Dalatias licha (Bonnaterre, 1788)	Species	Dalatiidae	Squaliformes	Chondrichthyes
Dasyatis brevicaudata (Hutton, 1875)	Species	Dasyatidae	Myliobatiformes	Chondrichthyes
Dasyatis thetidis Ogilby in Waite, 1899	Species	Dasyatidae	Myliobatiformes	Chondrichthyes
Deania calcea (Lowe, 1839)	Species	Centrophoridae	Squaliformes	Chondrichthyes
Diaphus sp.	Genus	Myctophidae	Myctophiformes	Actinopterygii
Diastobranchus capensis Barnard, 1923	Species	Synaphobranchidae	Anguilliformes	Actinopterygii
Dipturus innominatus (Garrick & Paul, 1974)	Species	Rajidae	Rajiformes	Chondrichthyes
Echeneis naucrates Linnaeus, 1758	Species	Echeneidae	Perciformes	Actinopterygii
Epinephelus daemelii (Günther, 1876)	Species	Serranidae	Perciformes	Actinopterygii
Eptatretus cf.cirrhatus (Forster in Bloch & Schneider, 1801)	Species	Myxinidae	Myxiniformes	Myxini
Eptatretus sp2	Species	Myxinidae	Myxiniformes	Myxini
Etelis coruscans Valenciennes, 1862	Species	Lutjanidae	Perciformes	Actinopterygii
Etmopterus baxteri Garrick, 1957	Species	Etmopteridae	Squaliformes	Chondrichthyes
Etmopterus lucifer Jordan & Snyder, 1902	Species	Etmopteridae	Squaliformes	Chondrichthyes
Etmopterus molleri (Whitley, 1939)	Species	Etmopteridae	Squaliformes	Chondrichthyes
Etmopterus sp.	Genus	Etmopteridae	Squaliformes	Chondrichthyes
Forsterygion flavonigrum Fricke & Roberts in Fricke, 1994	Species	Tripterygiidae	Perciformes	Actinopterygii
Forsterygion maryannae (Hardy, 1987)	Species	Tripterygiidae	Perciformes	Actinopterygii
Gadomus aoteanus McCann & McKnight, 1980	Species	Macrouridae	Gadiformes	Actinopterygii
Galeorhinus galeus (Linnaeus, 1758)	Species	Triakidae	Carcharhiniformes	Chondrichthyes
Gempylidae sp.	Family	Gempylidae	Perciformes	Actinopterygii
Genypterus blacodes (Forster in Bloch & Schneider, 1801)	Species	Ophidiidae	Ophidiiformes	Actinopterygii

Taxa	Level of identification	Family	Order	Class
Girella cyanea Macleay, 1881	Species	Kyphosidae	Perciformes	Actinopterygii
Gnathophis sp.	Genus	Congridae	Anguilliformes	Actinopterygii
Gollum attenuatus (Garrick, 1954)	Species	Pseudotriakidae	Carcharhiniformes	Chondrichthyes
Gorgasia japonica Abe, Miki & Asai, 1977	Species	Congridae	Anguilliformes	Actinopterygii
Gymnothorax berndti Snyder, 1904	Species	Muraenidae	Anguilliformes	Actinopterygii
Gymnothorax nubilus (Richardson, 1848)	Species	Muraenidae	Anguilliformes	Actinopterygii
Gymnothorax prasinus (Richardson, 1848)	Species	Muraenidae	Anguilliformes	Actinopterygii
Gymnothorax prionodon Ogilby, 1895	Species	Muraenidae	Anguilliformes	Actinopterygii
Gymnothorax prophyreus Ogilby, 1895	Species	Muraenidae	Anguilliformes	Actinopterygii
Halargyreus johnsoni Günther, 1862	Species	Moridae	Gadiformes	Actinopterygii
Halosaurus pectoralis McCulloch, 1926	Species	Halosauridae	Notacanthiformes	Actinopterygii
Halosaurus sp.	Genus	Halosauridae	Notacanthiformes	Actinopterygii
Helicolenus barathri Hector, 1875	Species	Scorpaenidae	Scorpaeniformes	Actinopterygii
Helicolenus percoides (Richardson, 1842)	Species	Scorpaenidae	Scorpaeniformes	Actinopterygii
Helicolenus sp.	Genus	Scorpaenidae	Scorpaeniformes	Actinopterygii
Hemerocoetes sp.	Genus	Percophidae	Perciformes	Actinopterygii
Heptranchias perlo (Bonnaterre, 1788)	Species	Hexanchidae	Hexanchiformes	Chondrichthyes
Hexanchus griseus (Bonnaterre, 1788)	Species	Hexanchidae	Hexanchiformes	Chondrichthyes
Hoplostethus atlanticus Collett, 1889	Species	Trachichthyidae	Beryciformes	Actinopterygii
Hoplostethus mediterraneus Cuvier in Cuvier & Valenciennes, 1839	Species	Trachichthyidae	Beryciformes	Actinopterygii
Hoplostethus sp.	Genus	Trachichthyidae	Beryciformes	Actinopterygii
Hydrolagus bemisi Didier, 2002	Species	Chimaeridae	Chimaeriformes	Chondrichthyes
Hydrolagus novaezelandiae (Fowler, 1911)	Species	Chimaeridae	Chimaeriformes	Chondrichthyes
Hydrolagus sp.	Genus	Chimaeridae	Chimaeriformes	Chondrichthyes
Hymenocephalus sp.	Genus	Macrouridae	Gadiformes	Actinopterygii
Hyperoglyphe antarctica (Carmichael, 1819)	Species	Centrolophidae	Perciformes	Actinopterygii
Hypoplectrodes sp.B	Species	Serranidae	Perciformes	Actinopterygii
Laemonema robustum Johnson, 1862	Species	Moridae	Gadiformes	Actinopterygii
Lagocephalus cf.cheesemanii (Clark, 1897)	Species	Tetraodontidae	Tetraodontiformes	Actinopterygii
Latridopsis ciliaris (Forster in Bloch & Schneider, 1801)	Species	Latridae	Perciformes	Actinopterygii
Latridopsis forsteri (Castelnau, 1872)	Species	Latridae	Perciformes	Actinopterygii
Latris lineata (Forster, 1801)	Species	Latridae	Perciformes	Actinopterygii
Lepidion microcephalus Cowper, 1956	Species	Moridae	Gadiformes	Actinopterygii
Lepidion schmidti Svetovidov, 1936	Species	Moridae	Gadiformes	Actinopterygii

Taxa	Level of identification	Family	Order	Class
Lepidoperca inornata Regan, 1914	Species	Serranidae	Perciformes	Actinopterygii
Lepidoperca sp.	Genus	Serranidae	Perciformes	Actinopterygii
Lepidopus caudatus (Euphrasen, 1788)	Species	Trichiuridae	Perciformes	Actinopterygii
Lepidorhynchus denticulatus (Richardson, 1846)	Species	Macrouridae	Gadiformes	Actinopterygii
Lepidorhynchus sp.	Genus	Macrouridae	Gadiformes	Actinopterygii
Liparidae sp.	Family	Liparidae	Scorpaeniformes	Actinopterygii
Lucigadus nigromaculatus (McCulloch, 1907)	Species	Macrouridae	Gadiformes	Actinopterygii
Lyconus sp.	Genus	Merlucciidae	Gadiformes	Actinopterygii
Macrouridae sp.	Family	Macrouridae	Gadiformes	Actinopterygii
Macrourus carinatus (Günther, 1878)	Species	Macrouridae	Gadiformes	Actinopterygii
Macruronus novaezelandiae (Hector, 1871)	Species	Merlucciidae	Gadiformes	Actinopterygii
Malacocephalus laevis (Lowe, 1843)	Species	Macrouridae	Gadiformes	Actinopterygii
Matanui profundum (Fricke & Roberts in Fricke, 1994)	Species	Tripterygiidae	Perciformes	Actinopterygii
Melanostigma gelatinosum Günther, 1881	Species	Zoarcidae	Perciformes	Actinopterygii
Microstomatidae sp.	Family	Microstomatidae	Osmeriformes	Actinopterygii
Mora moro (Risso, 1810)	Species	Moridae	Gadiformes	Actinopterygii
Moridae sp.	Family	Moridae	Gadiformes	Actinopterygii
Muraenolepis sp.	Genus	Muraenolepididae	Gadiformes	Actinopterygii
Mustelus n.sp.	Species	Triakidae	Carcharhiniformes	Chondrichthyes
Myctophidae sp.	Family	Myctophidae	Myctophiformes	Actinopterygii
Myliobatis tenuicaudatus Hector, 1877	Species	Myliobatidae	Myliobatiformes	Chondrichthyes
Nemadactylus douglasii (Hector, 1875)	Species	Cheilodactylidae	Perciformes	Actinopterygii
Nemadactylus macropterus (Forster, 1801)	Species	Cheilodactylidae	Perciformes	Actinopterygii
Nemadactylus n.sp.	Species	Cheilodactylidae	Perciformes	Actinopterygii
Neocyttus rhomboidalis Gilchrist, 1906	Species	Oreosomatidae	Zeiformes	Actinopterygii
Neomyxine biniplicata (Richardson & Jowett, 1951)	Species	Myxinidae	Myxiniformes	Myxini
Neomyxine sp1	Species	Myxinidae	Myxiniformes	Myxini
Nettastomatidae sp.	Family	Nettastomatidae	Anguilliformes	Actinopterygii
Nezumia sp.	Genus	Macrouridae	Gadiformes	Actinopterygii
Notacanthus sexspinis Richardson, 1846	Species	Notacanthidae	Notacanthiformes	Actinopterygii
Notolabrus cinctus (Hutton, 1877)	Species	Labridae	Perciformes	Actinopterygii
Notolabrus fucicola (Richardson, 1840)	Species	Labridae	Perciformes	Actinopterygii
Notolabrus inscriptus (Richardson, 1848)	Species	Labridae	Perciformes	Actinopterygii
Notophycis marginata (Günther, 1878)	Species	Moridae	Gadiformes	Actinopterygii
Notorynchus cepedianus (Péron, 1807)	Species	Hexanchidae	Hexanchiformes	Chondrichthyes

Taxa	Level of identification	Family	Order	Class
Notothenia angustata Hutton, 1875	Species	Nototheniidae	Perciformes	Actinopterygii
Notothenia microlepidota Hutton, 1875	Species	Nototheniidae	Perciformes	Actinopterygii
Odontaspis ferox (Risso, 1810)	Species	Odontaspididae	Lamniformes	Chondrichthyes
Ophisurus serpens (Linnaeus, 1758)	Species	Ophichthidae	Anguilliformes	Actinopterygii
Oreosomatidae sp.	Family	Oreosomatidae	Zeiformes	Actinopterygii
Pagrus auratus (Forster, 1801)	Species	Sparidae	Perciformes	Actinopterygii
Paralepididae sp.	Family	Paralepididae	Aulopiformes	Actinopterygii
Parapercis binivirgata (Waite, 1904)	Species	Pinguipedidae	Perciformes	Actinopterygii
Parapercis colias (Forster & Schneider, 1801)	Species	Pinguipedidae	Perciformes	Actinopterygii
Parapercis gilliesi (Hutton, 1879)	Species	Pinguipedidae	Perciformes	Actinopterygii
Paraulopus nigripinnis (Günther, 1878)	Species	Paraulopidae	Aulopiformes	Actinopterygii
Paraulopus okamurai Sato & Nakabo, 2002	Species	Paraulopidae	Aulopiformes	Actinopterygii
Paraulopus sp.	Genus	Paraulopidae	Aulopiformes	Actinopterygii
Parika scaber (Forster in Bloch & Schneider, 1801)	Species	Monacanthidae	Tetraodontiformes	Actinopterygii
Parmaturus sp.	Genus	Scyliorhinidae	Carcharhiniformes	Chondrichthyes
Parupeneus spilurus (Bleeker, 1854)	Species	Mullidae	Perciformes	Actinopterygii
Plectranthias bilaticlavia Paulin & Roberts, 1987	Species	Serranidae	Perciformes	Actinopterygii
Plectranthias maculicauda (Regan, 1914)	Species	Serranidae	Perciformes	Actinopterygii
Polymixia cf.busakhini Günther,1877	Species	Polymixiidae	Polymixiiformes	Actinopterygii
Polymixia sp.W	Species	Polymixiidae	Polymixiiformes	Actinopterygii
Polyprion americanus (Bloch & Schneider, 1801)	Species	Polyprionidae	Perciformes	Actinopterygii
Polyprion oxygeneios (Schneider & Forster in Bloch & Schneider, 1801)	Species	Polyprionidae	Perciformes	Actinopterygii
Proscymnodon plunketi (Waite, 1909)	Species	Somniosidae	Squaliformes	Chondrichthyes
Pseudocaranx georgianus (Cuvier in Cuvier & Valenciennes, 1833)	Species	Carangidae	Perciformes	Actinopterygii
Pseudocaranx sp.dentex (Bloch & Schneider 1801)	Species	Carangidae	Perciformes	Actinopterygii
Pseudolabrus luculentus (Richardson, 1848)	Species	Labridae	Perciformes	Actinopterygii
Pseudolabrus miles (Schneider & Forster, 1801)	Species	Labridae	Perciformes	Actinopterygii
Pseudophycis bachus (Forster, 1801)	Species	Moridae	Gadiformes	Actinopterygii
Pseudophycis barbata Günther, 1863	Species	Moridae	Gadiformes	Actinopterygii
Psychrolutes microporos Nelson, 1995	Species	Psychrolutidae	Scorpaeniformes	Actinopterygii
Pterygotrigla andertoni (Waite, 1910)	Species	Triglidae	Scorpaeniformes	Actinopterygii
Rajidae sp.	Family	Rajidae	Rajiformes	Chondrichthyes
Rexea solandri (Cuvier in Cuvier & Valenciennes, 1832)	Species	Gempylidae	Perciformes	Actinopterygii

Taxa	Level of identification	Family	Order	Class
Rhinochimaera pacifica (Mitsukuri, 1895)	Species	Rhinochimaeridae	Chimaeriformes	Chondrichthyes
Ruvettus pretiosus Cocco, 1829	Species	Gempylidae	Perciformes	Actinopterygii
Scolecenchelys castlei McCosker, 2006	Species	Ophichthidae	Anguilliformes	Actinopterygii
Scopelosaurus hamiltoni (Waite, 1916)	Species	Notosudidae	Aulopiformes	Actinopterygii
Scorpaena cardinalis Solander & Richardson in Richardson, 1842	Species	Scorpaenidae	Scorpaeniformes	Actinopterygii
Scorpaena papillosa (Schneider & Forster, 1801)	Species	Scorpaenidae	Scorpaeniformes	Actinopterygii
Scorpaena sp.	Genus	Scorpaenidae	Scorpaeniformes	Actinopterygii
Seriola lalandi Valenciennes in Cuvier & Valenciennes, 1833	Species	Carangidae	Perciformes	Actinopterygii
Seriola rivoliana Valenciennes in Cuvier & Valenciennes, 1833	Species	Carangidae	Perciformes	Actinopterygii
Seriolella brama (Günther, 1860)	Species	Centrolophidae	Perciformes	Actinopterygii
Simenchelys parasitica Gill in Goode & Bean, 1879	Species	Synaphobranchidae	Anguilliformes	Actinopterygii
Sphoeroides pachygaster (Müller & Troschel, 1848)	Species	Tetraodontidae	Tetraodontiformes	Actinopterygii
Squalus acanthias Linnaeus, 1758	Species	Squalidae	Squaliformes	Chondrichthyes
Squalus griffini Phillipps, 1931	Species	Squalidae	Squaliformes	Chondrichthyes
Squalus sp.	Genus	Squalidae	Squaliformes	Chondrichthyes
Squalus sp5	Species	Squalidae	Squaliformes	Chondrichthyes
Sternoptychidae sp.	Family	Sternoptychidae	Stomiiformes	Actinopterygii
Sternoptyx sp.	Genus	Sternoptychidae	Stomiiformes	Actinopterygii
Suezichthys cf.arquatus Russell, 1985	Species	Labridae	Perciformes	Actinopterygii
Suezichthys arquatus Russell, 1985	Species	Labridae	Perciformes	Actinopterygii
Suezichthys aylingi Russell, 1985	Species	Labridae	Perciformes	Actinopterygii
Suezichthys sp.	Genus	Labridae	Perciformes	Actinopterygii
Synaphobranchidae sp.	Family	Synaphobranchidae	Anguilliformes	Actinopterygii
Synaphobranchus cf.kaupii Johnson 1862	Species	Synaphobranchidae	Anguilliformes	Actinopterygii
Synaphobranchus affinis Günther, 1877	Species	Synaphobranchidae	Anguilliformes	Actinopterygii
Synaphobranchus sp.	Genus	Synaphobranchidae	Anguilliformes	Actinopterygii
Thamnaconus analis (Waite, 1904)	Species	Monacanthidae	Tetraodontiformes	Actinopterygii
Thyrsites atun (Euphrasen, 1791)	Species	Gempylidae	Perciformes	Actinopterygii
Torpedo fairchildi Hutton 1872	Species	Torpedinidae	Torpediniformes	Chondrichthyes
Torquigener altipinnis (Ogilby, 1891)	Species	Tetraodontidae	Tetraodontiformes	Actinopterygii
Trachurus sp.	Genus	Carangidae	Perciformes	Actinopterygii
Trachyrincus aphyodes McMillan, 1995	Species	Macrouridae	Gadiformes	Actinopterygii

**Taxa**	**Level of identification**	**Family**	**Order**	**Class**
Trachyrincus longirostris (Günther, 1878)	Species	Macrouridae	Gadiformes	Actinopterygii
Trachyrincus sp.	Genus	Macrouridae	Gadiformes	Actinopterygii
Tragulichthys pilatus Matsui & Rosenblatt, 1984	Species	Diodontidae	Tetraodontiformes	Actinopterygii
Tripterophycis gilchristi Boulenger, 1902	Species	Moridae	Gadiformes	Actinopterygii
Tripterophycis sp.	Genus	Moridae	Gadiformes	Actinopterygii
Upeneichthys lineatus (Bloch & Schneider, 1801)	Species	Mullidae	Perciformes	Actinopterygii
Zearaja nasutus (Banks in Müller & Henle, 1841)	Species	Rajidae	Rajiformes	Chondrichthyes
Zeidae sp.	Family	Zeidae	Zeiformes	Actinopterygii
Zenion leptolepis (Gilchrist & von Bonde, 1924)	Species	Zeniontidae	Zeiformes	Actinopterygii
Zeus faber Linnaeus, 1758	Species	Zeidae	Zeiformes	Actinopterygii

SUPPLEMENTARY TABLE S2. Species observed on two-hour baited remote underwater video deployments (stereo-BRUVS) ordered by locations (*n*=7) and depth strata (*n*=7) in New Zealand waters. Kermadec Islands (KER), Three Kings Islands (TKI), Great Barrier Island (GBI), White Island (WI), Kaikoura (KKA), Otago (OTA) and the Auckland Islands(AUC).

DEPTH STRATUM: 50 M

KER
Amphichaetodon howensis
Anampses sp.
Aulacocephalus temmincki
Bodianus unimaculatus
Canthigaster callisterna
Carcharhinus galapagensis
Chaetodon guentheri
Cheilodactylus francisi
Chromis dispila
Coris picta
Coris sandeyeri
Dasyatis brevicaudata
Dasyatis thetidis
Echeneis naucrates
Epinephelus daemelii
Girella cyanea
Gorgasia japonica
Gymnothorax nubilus
Mustelus n.sp.
Notolabrus inscriptus
Parupeneus spilurus
Pseudocaranx georgianus
Pseudolabrus luculentus
Seriola lalandi
Seriola rivoliana
Suezichthys arquatus
Suezichthys sp.
Thamnaconus analis
Torquigener altipinnis
Trachurus sp.

TKI
Caesioperca lepidoptera
Callanthias australis
Caprodon longimanus
Centroberyx affinis
Chromis sp.
Dasyatis brevicaudata
Forsterygion flavonigrum
Galeorhinus galeus
Hypoplectrodes sp.B
Myliobatis tenuicaudatus
Nemadactylus douglasii
Nemadactylus macropterus
Nemadactylus n.sp.
Notolabrus fucicola
Notorynchus cepedianus
Pagrus auratus
Parapercis colias
Parika scaber
Polyprion oxygeneios
Pseudocaranx georgianus
Pseudolabrus miles
Seriola lalandi
Squalus griffini
Suezichthys aylingi
Tragulichthys pilatus

GBI
Amphichaetodon howensis
Arripis xylabion
Bodianus flavipinnis
Bodianus unimaculatus
Caesioperca lepidoptera
Callanthias sp.
Caprodon longimanus
Cephaloscyllium isabellum
Chelidonichthys kumu
Chromis cf.abyssicola
Forsterygion flavonigrum
Galeorhinus galeus
Helicolenus percoides
Heptranchias perlo
Hypoplectrodes sp.B
Lepidopus caudatus
Nemadactylus douglasii
Nemadactylus macropterus
Nemadactylus n.sp.
Ophisurus serpens
Pagrus auratus
Parapercis colias
Parika scaber
Polyprion oxygeneios
Pseudocaranx georgianus
Pseudolabrus miles
Scorpaena cardinalis
Scorpaena sp.
Seriola lalandi
Thyrsites atun
Upeneichthys lineatus
Zearaja nasutus
Zeus faber

WI
Amphichaetodon howensis
Bodianus unimaculatus
Caesioperca lepidoptera
Callanthias australis
Canthigaster callisterna
Caprodon longimanus
Carcharhinus sp.
Centroberyx affinis
Cheilodactylus spectabilis
Chromis dispila
Coris sandeyeri
Dasyatis brevicaudata
Dasyatis thetidis
Forsterygion flavonigrum
Forsterygion maryannae
Gymnothorax berndti
Gymnothorax nubilus
Gymnothorax prasinus
Gymnothorax prionodon
Gymnothorax prophyreus
Latridopsis ciliaris
Latridopsis forsteri
Myliobatis tenuicaudatus
Nemadactylus douglasii
Nemadactylus macropterus
Notolabrus inscriptus
Pagrus auratus
Parika scaber
Pseudocaranx georgianus
Pseudocaranx sp.dentex
Pseudolabrus luculentus
Pterygotrigla andertoni
Scorpaena sp.
Seriola lalandi
Upeneichthys lineatus

KKA
Caesioperca lepidoptera
Callorhinchus milii
Cephaloscyllium isabellum
Coelorinchus biclinozonalis
Dipturus innominatus
Eptatretus cf.cirrhatus
Helicolenus percoides
Hydrolagus novaezelandiae
Nemadactylus macropterus
Neomyxine biniplicata
Notolabrus cinctus
Parapercis colias
Pseudolabrus miles
Pseudophycis bachus
Pseudophycis barbata
Pterygotrigla andertoni
Rajidae sp.
Scorpaena papillosa
Squalus acanthias

OTA
Cephaloscyllium isabellum
Chelidonichthys kumu
Dipturus innominatus
Hemerocoetes sp.
Nemadactylus macropterus
Parapercis colias
Polyprion oxygeneios
Squalus acanthias
Thyrsites atun
Zearaja nasutus

AUC
Bovichtus sp.
Forsterygion flavonigrum
Hemerocoetes sp.
Latris lineata
Matanui profundum
Notolabrus cinctus
Notothenia angustata
Notothenia microlepidota
Pseudophycis bachus

Thyrsites atun
Zearaja nasutus

DEPTH STRATUM: 100 M

KER
Amphichaetodon howensis
Bodianus unimaculatus
Canthigaster callisterna
Carcharhinus galapagensis
Dasyatis thetidis
Epinephelus daemelii
Gymnothorax nubilus
Lagocephalus cf.cheesemanii
Mustelus n.sp.
Nemadactylus n.sp.
Parapercis binivirgata
Pseudocaranx georgianus
Seriola lalandi
Squalus sp.
Suezichthys cf.arquatus

TKI
Caesioperca lepidoptera
Caprodon longimanus
Centroberyx affinis
Cephaloscyllium isabellum
Dasyatis brevicaudata
Eptatretus sp2
Galeorhinus galeus
Helicolenus percoides
Nemadactylus douglasii
Nemadactylus macropterus
Nemadactylus n.sp.
Notolabrus cinctus
Pagrus auratus
Parapercis binivirgata
Parapercis colias
Parapercis gilliesi
Parika scaber
Plectranthias maculicauda
Polyprion americanus
Polyprion oxygeneios
Pseudocaranx georgianus
Pseudolabrus miles
Seriola lalandi
Squalus griffini
Suezichthys aylingi
Thyrsites atun

GBI
Arripis xylabion
Bodianus unimaculatus
Caprodon longimanus
Centroberyx affinis
Cephaloscyllium isabellum
Cepola haasti
Chelidonichthys kumu
Dipturus innominatus
Eptatretus cf.cirrhatus
Forsterygion flavonigrum
Galeorhinus galeus
Helicolenus percoides
Nemadactylus macropterus
Neomyxine sp1
Pagrus auratus
Plectranthias maculicauda
Polyprion oxygeneios
Pseudocaranx georgianus
Seriola lalandi
Squalus griffini
Thyrsites atun
Zeus faber

WI
Amphichaetodon howensis
Bodianus unimaculatus
Caesioperca lepidoptera
Callanthias australis
Caprodon longimanus
Centroberyx affinis
Cephaloscyllium isabellum
Conger verreauxi
Dasyatis brevicaudata
Eptatretus cf.cirrhatus
Gymnothorax prasinus
Gymnothorax prionodon
Gymnothorax prophyreus
Latridopsis ciliaris
Myliobatis tenuicaudatus
Nemadactylus douglasii
Nemadactylus macropterus
Parapercis binivirgata
Parika scaber
Pseudocaranx georgianus
Scorpaena sp.
Seriola lalandi
Squalus griffini

KKA
Dipturus innominatus
Eptatretus cf.cirrhatus
Galeorhinus galeus
Helicolenus percoides
Hydrolagus novaezelandiae
Nemadactylus macropterus
Neomyxine biniplicata
Notolabrus cinctus
Parapercis colias
Polyprion oxygeneios
Pseudophycis bachus
Squalus acanthias
Thyrsites atun

OTA
Carcharodon carcharias
Cephaloscyllium isabellum
Colistium nudipinnis
Colistium sp.
Congiopodus sp.
Dipturus innominatus
Galeorhinus galeus
Nemadactylus macropterus
Parapercis colias
Pseudophycis bachus
Squalus acanthias
Thyrsites atun
Zearaja nasutus

AUC
Bovichtus sp.
Galeorhinus galeus
Hemerocoetes sp.
Matanui profundum
Notolabrus cinctus
Notothenia microlepidota
Pseudophycis bachus

DEPTH STRATUM: 300 M

KER
Centroberyx affinis
Epinephelus daemelii
Etelis coruscans
Helicolenus percoides
Nemadactylus n.sp.
Plectranthias bilaticlavia
Polyprion americanus
Rajidae sp.
Seriola lalandi
Sphoeroides pachygaster
Squalus sp.

TKI
Cirrhigaleus australis
Eptatretus sp2
Galeorhinus galeus
Helicolenus percoides
Hyperoglyphe antarctica
Lepidoperca inornata
Lepidoperca sp.
Nemadactylus n.sp.
Parapercis gilliesi
Plectranthias maculicauda
Polyprion americanus
Pterygotrigla andertoni
Seriola lalandi
Squalus griffini

GBI
Cephaloscyllium isabellum
Dipturus innominatus
Eptatretus cf.cirrhatus
Galeorhinus galeus
Gollum attenuatus
Helicolenus percoides
Helicolenus sp.
Microstomatidae sp.
Nemadactylus macropterus
Neomyxine sp1
Paraulopus nigripinnis
Paraulopus okamurai
Paraulopus sp.
Polyprion oxygeneios
Pterygotrigla andertoni
Rexea solandri
Squalus griffini
Zenion leptolepis

WI
Cephaloscyllium isabellum
Eptatretus cf.cirrhatus
Helicolenus barathri
Helicolenus percoides
Hydrolagus novaezelandiae
Hyperoglyphe antarctica
Myctophidae sp.
Nemadactylus macropterus
Parapercis gilliesi
Paraulopus nigripinnis
Paraulopus okamurai
Polyprion americanus
Pseudophycis barbata
Pterygotrigla andertoni
Rexea solandri
Squalus griffini

KKA
Bassanago bulbiceps
Bythaelurus dawsoni
Centroscymnus macracanthus
Coelorinchus bollonsi
Coelorinchus oliverianus
Dipturus innominatus
Eptatretus cf.cirrhatus
Etmopterus molleri
Genypterus blacodes
Helicolenus barathri
Helicolenus percoides
Hexanchus griseus
Hydrolagus novaezelandiae
Lepidorhynchus denticulatus
Macrouridae sp.
Macruronus novaezelandiae
Melanostigma gelatinosum
Neomyxine biniplicata
Neomyxine sp1
Polyprion oxygeneios
Pseudophycis bachus
Squalus acanthias
Thyrsites atun
Torpedo fairchildi
Zearaja nasutus

OTA
Bassanago bulbiceps
Cephaloscyllium isabellum
Coelorinchus aspercephalus
Coelorinchus fasciatus
Dipturus innominatus
Helicolenus percoides
Hydrolagus novaezelandiae
Lepidorhynchus denticulatus
Macrouridae sp.
Macruronus novaezelandiae
Myctophidae sp.
Notophycis marginata
Rajidae sp.
Seriolella brama
Squalus acanthias
Thyrsites atun
Zearaja nasutus

AUC
Coelorinchus aspercephalus
Coelorinchus sp.
Notothenia microlepidota
Pseudophycis bachus

DEPTH STRATUM: 500 M

KER
Beryx splendens
Cephaloscyllium sp.B
Diaphus sp.
Etmopterus molleri
Helicolenus sp.
Hyperoglyphe antarctica
Macrouridae sp.
Malacocephalus laevis
Myctophidae sp.
Paraulopus okamurai
Polymixia cf.busakhini
Polyprion americanus
Squalus sp.

TKI
Cirrhigaleus australis
Eptatretus sp2
Etmopterus sp.
Gollum attenuatus
Helicolenus barathri
Hyperoglyphe antarctica
Macrouridae sp.
Notophycis marginata
Paraulopus okamurai
Plectranthias maculicauda
Polyprion americanus
Squalus griffini

GBI
Bassanago bulbiceps
Benthodesmus sp.
Capromimus abbreviatus
Centriscops humerosus
Cephaloscyllium isabellum
Dipturus innominatus
Eptatretus cf.cirrhatus
Etmopterus molleri
Genypterus blacodes
Gollum attenuatus
Helicolenus barathri
Hoplostethus mediterraneus
Hyperoglyphe antarctica
Macruronus novaezelandiae
Mora moro
Myctophidae sp.
Rexea solandri
Scolecenchelys castlei
Squalus griffini
Squalus sp.
Squalus sp5
Sternoptychidae sp.
Tripterophycis sp.

WI
Benthodesmus sp.
Beryx decadactylus
Capromimus abbreviatus
Centriscops humerosus
Coelorinchus mystax
Cyttus novaezealandiae
Dalatias licha
Dipturus innominatus
Eptatretus cf.cirrhatus
Genypterus blacodes
Helicolenus barathri
Hyperoglyphe antarctica
Lepidorhynchus denticulatus
Macrouridae sp.
Macruronus novaezelandiae
Mora moro
Myctophidae sp.
Paraulopus okamurai
Rexea solandri
Squalus acanthias
Squalus sp5
Tripterophycis gilchristi

KKA
Bassanago bulbiceps
Centriscops humerosus
Centroscymnus macracanthus
Coelorinchus bollonsi
Coelorinchus oliverianus
Deania calcea
Eptatretus cf.cirrhatus
Genypterus blacodes
Helicolenus barathri
Hyperoglyphe antarctica
Lepidorhynchus denticulatus
Macrouridae sp.
Macruronus novaezelandiae
Mora moro
Proscymnodon plunketi
Zeidae sp.

OTA
Bassanago bulbiceps
Coelorinchus fasciatus
Coelorinchus oliverianus
Dalatias licha
Dipturus innominatus
Eptatretus cf.cirrhatus
Etmopterus baxteri
Genypterus blacodes
Helicolenus barathri
Hyperoglyphe antarctica
Lepidorhynchus denticulatus
Macruronus novaezelandiae
Mora moro
Myctophidae sp.
Proscymnodon plunketi
Seriolella brama

AUC
Coelorinchus fasciatus
Hexanchus griseus
Lepidorhynchus denticulatus
Notophycis marginata
Proscymnodon plunketi

DEPTH STRATUM: 700 M

KER
Centrophorus harrissoni
Diaphus sp.
Laemonema robustum
Malacocephalus laevis
Myctophidae sp.
Polymixia cf.busakhini
Polymixia sp.W
Ruvettus pretiosus
Squalus sp.

TKI
Bassanago bulbiceps
Cirrhigaleus australis
Deania calcea
Eptatretus cf.cirrhatus
Eptatretus sp2
Etmopterus molleri
Genypterus blacodes
Helicolenus barathri
Hexanchus griseus
Hyperoglyphe antarctica
Mora moro
Polyprion americanus
Rexea solandri
Squalus griffini
Squalus sp.
Squalus sp5

GBI
Bassanago bulbiceps
Bathyraja shuntovi
Dalatias licha
Deania calcea
Dipturus innominatus
Eptatretus cf.cirrhatus
Gempylidae sp.
Genypterus blacodes
Hoplostethus mediterraneus
Hyperoglyphe antarctica
Lepidorhynchus sp.
Macrouridae sp.
Macruronus novaezelandiae
Mora moro
Myctophidae sp.
Neomyxine sp1
Proscymnodon plunketi
Rexea solandri
Scolecenchelys castlei
Scopelosaurus hamiltoni
Squalus sp5

WI
Alepocephalidae sp.
Bassanago bulbiceps
Coelorinchus sp.
Dalatias licha
Deania calcea
Eptatretus cf.cirrhatus
Etmopterus molleri
Etmopterus sp.
Gempylidae sp.
Genypterus blacodes
Helicolenus barathri
Hoplostethus sp.
Hydrolagus sp.
Hyperoglyphe antarctica
Laemonema robustum
Lepidorhynchus denticulatus
Macrouridae sp.
Macruronus novaezelandiae
Mora moro
Myctophidae sp.
Neomyxine sp1
Odontaspis ferox
Paralepididae sp.
Rexea solandri
Ruvettus pretiosus
Synaphobranchidae sp.
Synaphobranchus affinis
Synaphobranchus sp.

KKA
Bassanago bulbiceps
Centriscops humerosus
Centroscymnus macracanthus
Centroselachus crepidater
Coelorinchus innotabilis
Deania calcea
Eptatretus cf.cirrhatus
Etmopterus baxteri
Etmopterus molleri
Genypterus blacodes
Helicolenus barathri
Hyperoglyphe antarctica
Lepidorhynchus denticulatus
Macrouridae sp.
Macruronus novaezelandiae
Mora moro
Myctophidae sp.
Notacanthus sexspinis
Proscymnodon plunketi
Sternoptyx sp.

OTA
Allocyttus niger
Bassanago bulbiceps
Centroscymnus owstoni
Chimaeridae sp.
Coelorinchus fasciatus
Coryphaenoides subserrulatus
Diastobranchus capensis
Dipturus innominatus
Etmopterus baxteri
Genypterus blacodes
Gnathophis sp.
Halargyreus johnsoni
Hydrolagus bemisi
Lepidorhynchus denticulatus
Lucigadus nigromaculatus
Macrouridae sp.
Macruronus novaezelandiae
Mora moro
Neocyttus rhomboidalis
Oreosomatidae sp.
Proscymnodon plunketi
Seriolella brama
Simenchelys parasitica

AUC
Bassanago bulbiceps
Centroscymnus owstoni
Coelorinchus fasciatus
Coelorinchus oliverianus
Etmopterus baxteri
Genypterus blacodes
Lepidorhynchus denticulatus
Macrouridae sp.
Macruronus novaezelandiae
Mora moro
Notacanthus sexspinis
Proscymnodon plunketi

DEPTH STRATUM: 900 M

KER
Bathygadus cottoides
Bathypterois longifilis
Benthodesmus sp.
Centrophorus harrissoni
Centrophorus sp.
Coelorinchus kermadecus
Hexanchus griseus
Macrouridae sp.
Malacocephalus laevis
Mora moro
Myctophidae sp.
Nezumia sp.
Squalus sp.
Synaphobranchus affinis

TKI
Bassanago bulbiceps
Centroscymnus owstoni
Deania calcea
Diastobranchus capensis
Eptatretus cf.cirrhatus
Etmopterus baxteri
Halosaurus pectoralis
Hoplostethus mediterraneus
Hymenocephalus sp.
Macrouridae sp.
Mora moro
Moridae sp.
Proscymnodon plunketi
Simenchelys parasitica
Synaphobranchus affinis

GBI
Bassanago bulbiceps
Bathyraja shuntovi
Benthodesmus sp.
Centroscymnus owstoni
Coelorinchus mycterismus
Coelorinchus sp.
Coryphaenoides serrulatus
Dalatias licha
Deania calcea
Diastobranchus capensis
Genypterus blacodes
Macrouridae sp.
Mora moro
Myctophidae sp.
Neomyxine sp1
Rexea solandri
Ruvettus pretiosus
Simenchelys parasitica
Synaphobranchus affinis
Trachyrincus aphyodes

WI
Apristurus sp.
Bassanago bulbiceps
Benthodesmus sp.
Centroscymnus owstoni
Coelorinchus sp.
Coryphaenoides serrulatus
Dalatias licha
Deania calcea
Diastobranchus capensis
Gadomus aoteanus
Hoplostethus atlanticus
Hoplostethus sp.
Macrouridae sp.
Macruronus novaezelandiae
Mora moro
Myctophidae sp.
Neomyxine biniplicata
Neomyxine sp1
Rexea solandri
Ruvettus pretiosus
Simenchelys parasitica
Synaphobranchidae sp.
Synaphobranchus affinis
Trachyrincus aphyodes

KKA
Alepocephalidae sp.
Bassanago bulbiceps
Brotulotaenia nigra
Centriscops humerosus
Centroscymnus coelolepis
Centroscymnus macracanthus
Centroscymnus owstoni
Centroselachus crepidater
Coelorinchus innotabilis
Coelorinchus oliverianus
Coryphaenoides serrulatus
Coryphaenoides subserrulatus
Deania calcea
Diastobranchus capensis
Etmopterus baxteri
Etmopterus sp.
Hydrolagus bemisi
Hyperoglyphe antarctica
Lepidion microcephalus
Lepidorhynchus denticulatus
Macruronus novaezelandiae
Mora moro
Neomyxine biniplicata
Notacanthus sexspinis
Proscymnodon plunketi
Simenchelys parasitica
Sternoptyx sp.
Synaphobranchus affinis
Trachyrincus aphyodes

OTA
Allocyttus niger
Bassanago bulbiceps
Centroscymnus owstoni
Coryphaenoides subserrulatus
Diastobranchus capensis
Etmopterus baxteri
Genypterus blacodes
Hoplostethus atlanticus
Lepidion microcephalus
Lepidorhynchus denticulatus
Macrouridae sp.
Macruronus novaezelandiae
Mora moro
Oreosomatidae sp.
Proscymnodon plunketi
Psychrolutes microporos
Rhinochimaera pacifica
Seriolella brama
Simenchelys parasitica
Synaphobranchidae sp.

AUC
Bassanago bulbiceps
Centroscymnus owstoni
Coryphaenoides subserrulatus
Deania calcea
Diastobranchus capensis
Etmopterus baxteri
Helicolenus percoides
Lepidion schmidti
Lepidorhynchus denticulatus
Lyconus sp.
Macrourus carinatus
Macruronus novaezelandiae
Mora moro
Notacanthus sexspinis
Proscymnodon plunketi
Simenchelys parasitica
Trachyrincus sp.

DEPTH STRATUM: 1200 M

KER
Bathygadus cottoides
Centroscymnus owstoni
Coelorinchus kermadecus
Coryphaenoides murrayi
Coryphaenoides rudis
Coryphaenoides serrulatus
Diastobranchus capensis
Etmopterus sp.
Liparidae sp.
Nezumia sp.
Odontaspis ferox
Simenchelys parasitica
Synaphobranchus affinis

TKI
Alepocephalus australis
Centroscymnus owstoni
Diastobranchus capensis
Etmopterus baxteri
Gadomus aoteanus
Lepidion microcephalus
Macrouridae sp.
Parmaturus sp.
Proscymnodon plunketi
Rhinochimaera pacifica
Simenchelys parasitica
Synaphobranchus affinis

GBI
Antimora rostrata
Bathygadus cottoides
Centroscymnus owstoni
Chauliodus sloani
Coelorinchus acanthiger
Coelorinchus kermadecus
Coryphaenoides serrulatus
Diastobranchus capensis
Etmopterus baxteri
Gadomus aoteanus
Halosaurus sp.
Lepidion microcephalus
Macrouridae sp.
Mora moro
Myctophidae sp.
Neomyxine sp1
Nettastomatidae sp.
Oreosomatidae sp.
Parmaturus sp.
Ruvettus pretiosus
Simenchelys parasitica
Synaphobranchus cf.kaupii
Synaphobranchus affinis
Trachyrincus aphyodes

WI

KKA
Allocyttus sp.
Centroscymnus owstoni
Coryphaenoides subserrulatus
Diastobranchus capensis
Etmopterus baxteri
Etmopterus lucifer
Etmopterus sp.
Lepidion microcephalus
Macrouridae sp.
Proscymnodon plunketi
Simenchelys parasitica
Synaphobranchus affinis
Trachyrincus aphyodes
Trachyrincus longirostris

OTA
Amblyraja cf.hyperborea
Antimora rostrata
Coelorinchus bollonsi
Coryphaenoides subserrulatus
Diastobranchus capensis
Etmopterus baxteri
Lepidion microcephalus
Lepidorhynchus denticulatus
Macrouridae sp.
Moridae sp.
Muraenolepis sp.
Simenchelys parasitica

AUC

Supplementary Video S3. Video footage of communities of fishes observed within each depth stratum. Map was created using the R software[1]. Bathymetry source: Depth contour polyline (Hydro, 1:350k - 1:1,500k), Land Information New Zealand, Crown Copyright Reserved. Land data source: NZ Coastlines (Topo, 1:50k), Land Information New Zealand, Crown Copyright Reserved.

SUPPLEMENTARY FIGURE S4. Species accumulation curves for fishes sampled within each depth stratum (999 permutations) from two-hour baited remote underwater video deployments (stereo-BRUVS) across 7 locations in New Zealand waters (Kermadec Islands, Three Kings Islands, Great Barrier Island, White Island, Kaikoura, Otago and the Auckland Islands).

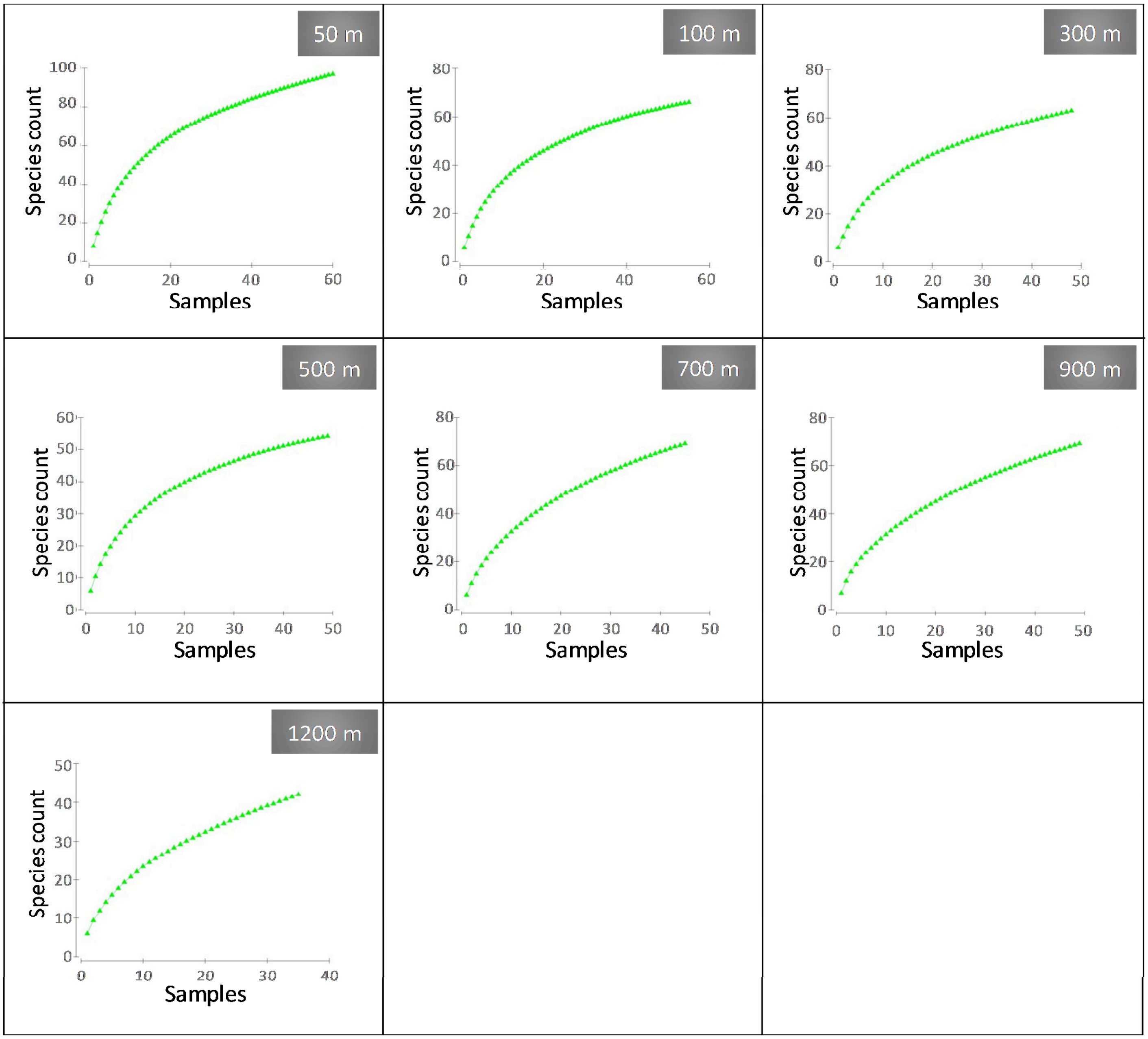

SUPPLEMENTARY FIGURE S5. Species richness of fishes as a function of depth of sampling (7 levels: 50, 100, 300, 500, 700, 900, 1200 m), as obtained from two-hour baited remote underwater video deployments (stereo-BRUVS) across 7 locations in New Zealand waters (Kermadec Islands, Three Kings Islands, Great Barrier Island, White Island, Kaikoura, Otago and the Auckland Islands). (A) Average sample species richness (± SE) for all locations; (B) to (H) Average sample species richness (± SE) per location (alpha diversity) and species richness for each location x depth stratum combination (gamma diversity).

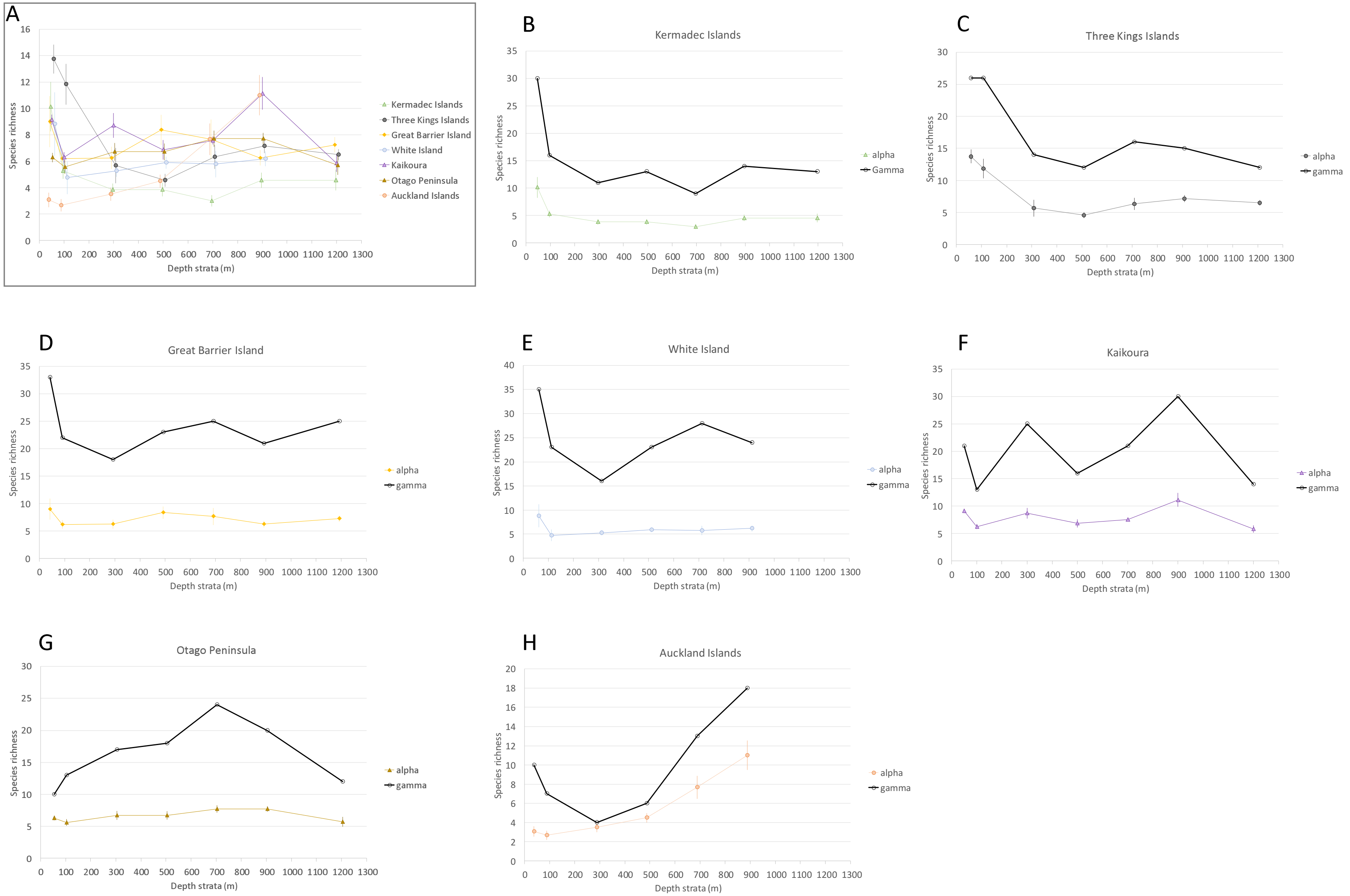

SUPPLEMENTARY METHOD S6. R code[1] for calculating *pseudo* multivariate components of variation in crossed designs.

```
#########################################################################################
#
# Function in R code for calculating the components of variation
# for multivariate data on the basis of the Jaccard measure in response to
# a two-factor design (factors A and B), including bootstrap
# confidence intervals with empirical bias-correction.
#
# Input consists of the following object:
#
# data.df = A data frame object of dimension N rows (sample units) by (p+2) columns
#           with the following structure:
#
#    column 1 = factor A with groups whose variation is to be estimated,
#               separately within each level factor B;
#    column 2 = factor B specifying groups within which components are to be estimated;
#    columns 3 to (p+2) = multivariate data of dimension N rows (sample units)
#        by p columns (variables);
#
# Note: The order of the factor levels will be taken by the existing
#       "levels" of each factor on input.
#
# The function allows for unbalanced designs
# (missing sample units per cell or missing cells).
#
# Output yields a single object:
# results = summary table of results, with the estimated components of variation
#           due to factor A and due to the residual (as separate rows),
#           separately within each level of factor B (columns)
#           along with the lower and upper 95% CI based on the bias-adjusted
#           bootstrap for each of these (additional rows).
#
# plus (optionally) four separate *.CSV files (by specifying outcsv = "T"):
# bootA   = values under bootstrapping for the estimated component of
#               variation due to factor A (within different levels
#               of factor B, given in separate columns)
# bootRes = values under bootstrapping for the estimated residual component
#               (within different levels of factor B, given in
#                separate columns)
# bias    = table of values obtained for the bias in the bootstrap for each component
# results = summary table as above, given in a csv file.
#
#
#########################################################################################
#
comp.var = function(data.df, nboot = 1000, outcsv = "F") {

   A = factor(data.df[,1]); B = factor(data.df[,2])
   Y = data.df[,-c(1,2)]
   p = dim(Y)[2] # number of variables in the multivariate data
   a = length(sort(unique(A))) # max number of levels of A
   b = length(sort(unique(B))) # max number of levels of B
   obs.MS_A = rep(0,b)
   obs.MS_Res = rep(0,b)
   var.A = rep(0,b)
   bias = matrix(rep(0,2*b), ncol = b, nrow = 2)
   colnames(bias) <- levels(B)
   rownames(bias) <- c("bias.var.A","bias.var.Res")

   Lower.A = rep(0,b)
   Upper.A = rep(0,b)
   Lower.Res = rep(0,b)
   Upper.Res = rep(0,b)

   var.A.boot = matrix(rep(0,b*nboot),ncol = b, nrow = nboot)
   var.Res.boot = matrix(rep(0,b*nboot),ncol = b, nrow = nboot)

# Loop through and calculate component of variation for factor A
# separately for each level of factor B
   for (j in 1:b) {
# Get the subset of data corresponding to one level of factor B
      Y.subset = subset(data.df, subset = (B == levels(B)[j]),
                               select = 3:(p+2))
# Note: within each subset, we have:
    # g = number of groups
    # n = vector of length g, with sample sizes per group
    # N = sum(n), the total number of observations in the subset of data
    # p = the number of variables

      N = dim(Y.subset)[1]
      A.factor = factor(A)[B == levels(B)[j]]
# The following deals with situations where the subset of data in question does not
# include all levels of factor A.
      my.table = as.data.frame(table(A.factor))
      n.prelim = my.table$Freq
      nonzero.levels = as.vector(my.table$A.factor[n.prelim!=0])
      A.factor = factor(A.factor, levels = nonzero.levels)
      n = table(A.factor)
      g = length(n)

# Calculations that can be done more efficiently outside of the bootstrapping loop:
# For the PERMANOVA partitioning
```

```
      X.full = model.matrix(~A.factor)            #model matrix
      X.reduced = X.full[,1]                   #model with intercept only

# Hat matrices, df and the constant to be used for ANOVA estimators of components
      H.reduced = X.reduced %*% solve(t(X.reduced)%*%X.reduced)%*%t(X.reduced)
      H.full = X.full %*% solve(t(X.full)%*%X.full)%*%t(X.full)
      H = H.full - H.reduced

      I = diag(N)                              #Identity matrix
      Hres = (I - H.full)                      #for calculating residual SS
      ones = rep(1, N)                         #for calculating G
      df.num = (g - 1)
      df.denom = (N - g)
      constant = (N - sum(n^2)/N ) / (g - 1)  # e.g., see Searle et al. (1992) "Variance components" p. 428

# Distance matrix, ranked distance matrix, Gower's G matrix
# And residualized G matrix
      D = dist(Y.subset, method="binary")   # Note! This is the Jaccard dissimilarity measure.
      dmat = as.matrix(D^2)
      alpha = - 0.5 * dmat
      G = alpha - apply(alpha,1,mean) %o% ones - ones %o% apply(alpha,2,mean) + mean(alpha)

# Observed values for the mean squares (MS) for each of factor A and the Residual
      obs.MS_A[j] = (sum(G * t(H))/df.num)  # Observed among-group mean square
      obs.MS_Res[j] = (sum(G * t(Hres))/df.denom)  # Observed residual mean square

# Observed value for the component of variation for factor A
      var.A[j] = ( obs.MS_A[j] - obs.MS_Res[j] ) / constant # ANOVA estimator as in Searle et al. (1992), p. 
428

# Start the bootstrap loop
      index=1:N

      # Get a bootstrap sample with replacement of the correct sample size (n) for each group

      for (iboot in 1:nboot) {
         icount=0
         take=rep(0,N)
         for (i in 1:g) {
            icount = icount+n[i]
            begin = icount-n[i]+1
            take[begin:icount] = sample(index[begin:icount],n[i],replace=T)
            }
         G.boot = G[take,take]
         var.A.boot[iboot,j] = ( (sum(G.boot * t(H))/df.num) - (sum(G.boot * t(Hres))/df.denom) ) / constant
         var.Res.boot[iboot,j] = (sum(G.boot * t(Hres))/df.denom)

         } # close bootstrap loop

# Note that, under bootstrapping, the bias value for the estimation of var.D is negative,
# because the bias value for the residual mean square under separate-sample bootstrapping is positive
# (i.e. bootstrap values of residual mean square are smaller than the original, on average).

      bias[1, j] = var.A[j] - mean(var.A.boot[,j])
      bias[2, j] = obs.MS_Res[j] - mean(var.Res.boot[,j])

# Here are histograms of the bootstrap estimates, for example, along with the observed value
#      par(mfrow=c(1,2))
#      hist(var.A.boot[,j], main = paste("Var Component, A",levels(B)[j], sep = " "))
#      abline(v = var.A[j], col="red")
#      hist(var.Res.boot[,j], main = paste("Residual Component",levels(B)[j], sep = " "))
#      abline(v = obs.MS_Res[j], col="red")

# Now get the quantiles and adjust for bias

      Lower.A[j] = quantile(var.A.boot[,j], probs = 0.025) + bias[1, j]
      Upper.A[j] = quantile(var.A.boot[,j], probs = 0.975) + bias[1, j]
      Lower.Res[j] = quantile(var.Res.boot[,j], probs = 0.025) + bias[2, j]
      Upper.Res[j] = quantile(var.Res.boot[,j], probs = 0.975) + bias[2, j]

   } # close the loop being run on each level of factor B

# check out the results

   var.Res = obs.MS_Res
   results = rbind(Lower.A,var.A,Upper.A,Lower.Res,var.Res,Upper.Res)
   colnames(results) <- levels(B)
   colnames(var.A.boot) <- colnames(var.Res.boot) <- colnames(results)

# Write results to a file for future reference
   if(outcsv=="T") {
      write.csv(var.A.boot, file = "bootA.csv")
      write.csv(var.Res.boot, file = "bootRes.csv")
      write.csv(bias, file = "bias.csv")
      write.csv(results, file = "results.csv")
      }

   return(results)

   }   # end of function
###############################################################################################


# Usage
```

```
comp.var(data.df,nboot = 1000,outcsv="T")
```

References

1 R: A language and environment for statistical computing (R Foundation for Statistical Computing, Vienna, 2016).
